# Peutz-Jeghers syndrome with gastric-type mucinous endocervical adenocarcinoma and sex-cord tumor with annular tubules: A case report

**DOI:** 10.3389/fmed.2023.1094839

**Published:** 2023-03-21

**Authors:** Xuanyan Li, Yue Qi, Wenwen Zhang, Yang Rao, Na Zhang, Pengpeng Qu

**Affiliations:** ^1^Department of Gynecological Oncology, Tianjin Central Hospital Gynecology Obstetrics, Tianjin, China; ^2^Nankai University School of Medicine, Nankai University, Tianjin, China; ^3^Clinical College of Central Gynecology and Obstetrics, Tianjin Medical University, Tianjin, China

**Keywords:** Peutz-Jeghers syndrome, gastric-type mucinous endocervical adenocarcinoma, sex-cord tumor with annular tubules, diagnosis, treatment

## Abstract

Peutz-Jeghers syndrome (PJS) is a rare autosomal dominant genetic disorder characterized by mucocutaneous pigmentation and multiple hamartomatous polyps in the gastrointestinal tracts. About 11% of female PJS patients are diagnosed with Gastric-type endocervical adenocarcinoma (G-EAC) and about one third have a sex-cord tumor with annular tubules (SCTATs). Gastric-type endocervical adenocarcinoma is a special subtype of cervical adenocarcinoma which accounts for only 1–3%. Here we report a rare case of a 31-year-old woman affected with G-EAC and SCTAT accompanied by PJS. After surgery, we followed up for 5 years without recurrence.

## 1. Introduction

Peutz-Jeghers syndrome (PJS), also known as familial mucocutaneous hyperpigmented gastrointestinal polyposis, is a rare autosomal dominant disorder characterized by multiple hamartoma polyps in the oral cavity, skin, and mucous membranes, as well as by multiple hamartoma polyps in the gastrointestinal tract. The estimated incidence ranges from 1/50,000 to 1/250,000 (1). According to previous reports, 11–17% of women with PJS have gastric-type endocervical adenocarcinoma (G-EAC) (2) and sex-cord tumors with annular tubules (SCTATs).

In the 2020 World Health Organization(WHO) classification of female genital tumors, endocervical adenocarcinomas are subclassified into human papillomavirus (HPV)-associated and HPV-independent types based on their distinct etiology and clinical characteristics (3). G-EAC, a mucinous adenocarcinoma with gastric differentiation, is the most common type of HPV-independent cervical adenocarcinoma (4). The 2020 NCCN guidelines have indicated that the proportion of G-EAC in cervical adenocarcinomas has gradually increased to 10–15% (5). In addition, the disease is highly malignant and aggressive, often resulting in a poor prognosis.

Sex-cord tumor with annular tubule (SCTAT) is a special pathological type of sex-cord stromal tumor, accounting for approximately 5% of sex-cord stromal tumors (6). SCTATs of the ovaries are rare, but are commonly associated with PJS. According to statistics, about 36% of women with PJS have SCTAT, which is the most common ovarian tumor in PJS (7). SCTAT with PJS is mostly benign and has a good prognosis; SCTAT without PJS is mostly unilateral and large, and about 20% are malignant tumors (8).

## 2. Case description

**Figure 1:**
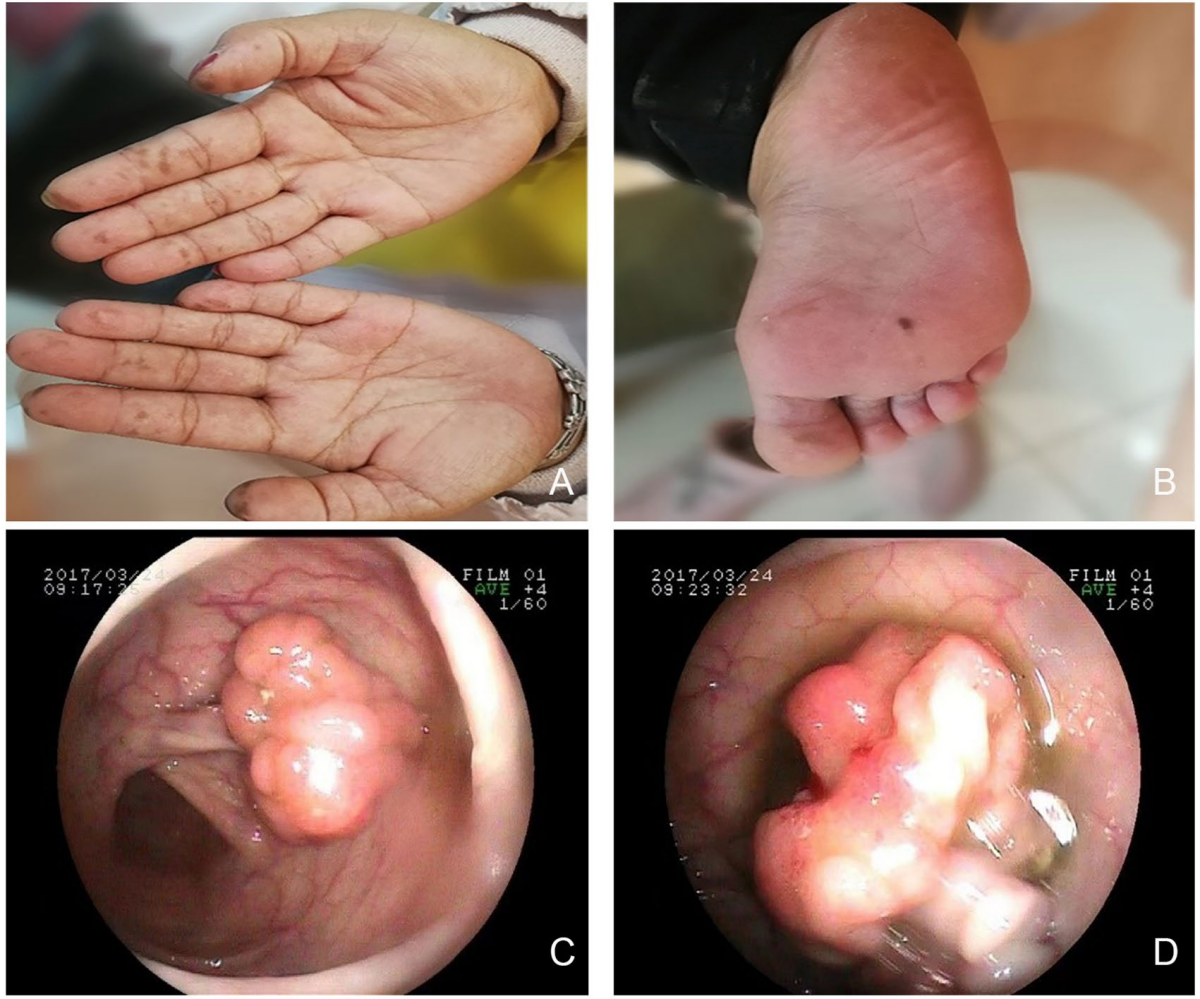
Multiple hyperpigmentations were visible on the skin. **(A)** hands. **(B)** right foot. Gastrointestinal endoscopy revealed multiple polyps. **(C)** small bowel. **(D)** ileum.

A 31-year-old nulliparous woman with increased vaginal discharge for 9 years visited our institution. It is worth noting that the patient underwent partial small bowel resection due to recurrent intussusception 7 years prior. Pathological examination revealed multiple adenomatous polyps of the small bowel. In addition, several melanin pigments were observed on the tips of the patient’s fingers, lips, and feet (Figures 1A,B). The patient’s parents died of other medical conditions, and have not received gastrointestinal tract examinations previously. But the patient’s brother and nephew were diagnosed with multiple intestinal polyps recently. Considering the patient’s medical history, we recommended she undergo gastrointestinal endoscopy. We found several adenomatous polyps of different sizes scattered throughout the gastrointestinal tract (Figures 1C,D). The largest polyp measured approximately 2 cm in diameter. A total of 15 polyps were excised and shown to be tubular adenomas, and some glands showed mild to moderate dysplasia. According to the World Health Organization (WHO) clinico-pathological criteria for diagnosing PJS, there were more than three polyps showing a Peutz-Jegher polyp (a smooth muscle core arising from the muscular mucosae and ramifying into the substance of the polyp like the branches of a tree). Due to these findings, the patient was diagnosed with PJS.

**Figure 2:**
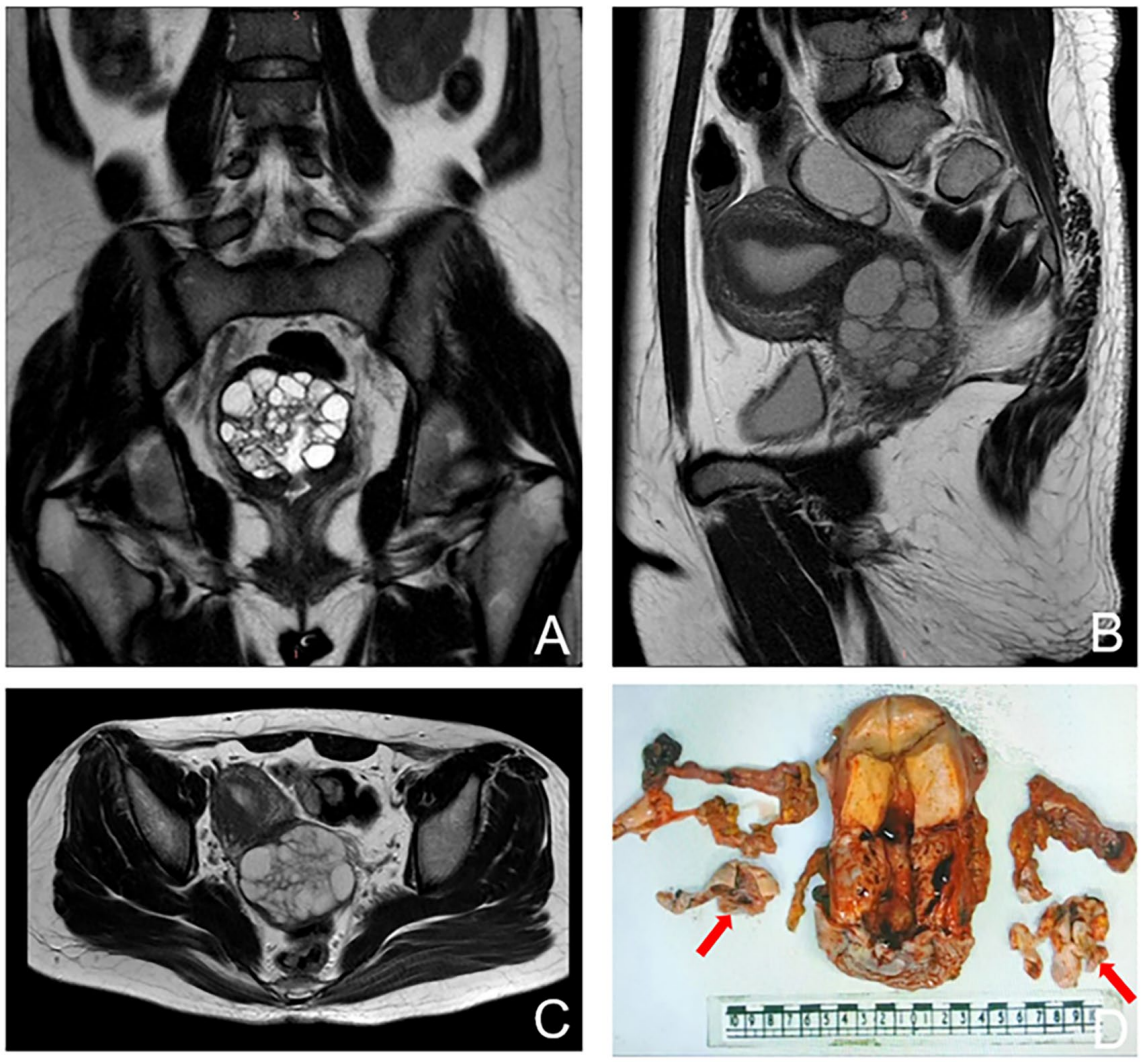
Magnetic resonance imaging (MRI) images showing an enlargement of the cervix with multiple cystic lesions. **(A)** coronal plane. **(B)** sagittal plane. **(C)** transverse plane. **(D)** Gross features of the uterus showing bulging cervix, fallopian, and ovary. The arrows show bilateral ovaries.

Pelvic examination revealed clear mucoid vaginal discharge. The surface of the cervix was smooth but firm with multiple nabothian cysts. Sonographic findings showed that the cervix was slightly enlarged with uneven recovery, and there was a cystic mass (37 × 40 × 36 mm) in the right adnexa. The results of the human papillomavirus (HPV) and liquid-based cytologic tests were normal. Serum levels of CA125 and CA199 were within normal limits. Pelvic magnetic resonance imaging (MRI) demonstrated that the cervix was enlarged with a multilocular cystic lesion, and the right ovary was cystic. The mass was confined to the cervix without parametrial involvement, and there was no evidence of metastatic lymphadenopathy (Figures 2A–C). Colposcopy-guided cervical biopsy and hysteroscopy were performed, but a diagnosis could not be made. Finally, cervical biopsy under ultrasonography confirmed a G-EAC. Before surgery, clinical stage IB2 was diagnosed in accordance with the International Federation of Gynecology and Obstetrics criteria. In addition, the patient was infertile. Therefore, diagnostic conization of the cervix was performed to confirm tumor size and depth before radical surgery. Unfortunately, the residual cervical apex and cutting-edge histopathological examination revealed gastric-type mucinous carcinomas. The patient underwent laparoscopic radical hysterectomy, bilateral salpingo-oophorectomy, and bilateral pelvic node dissection.

**Figure 3:**
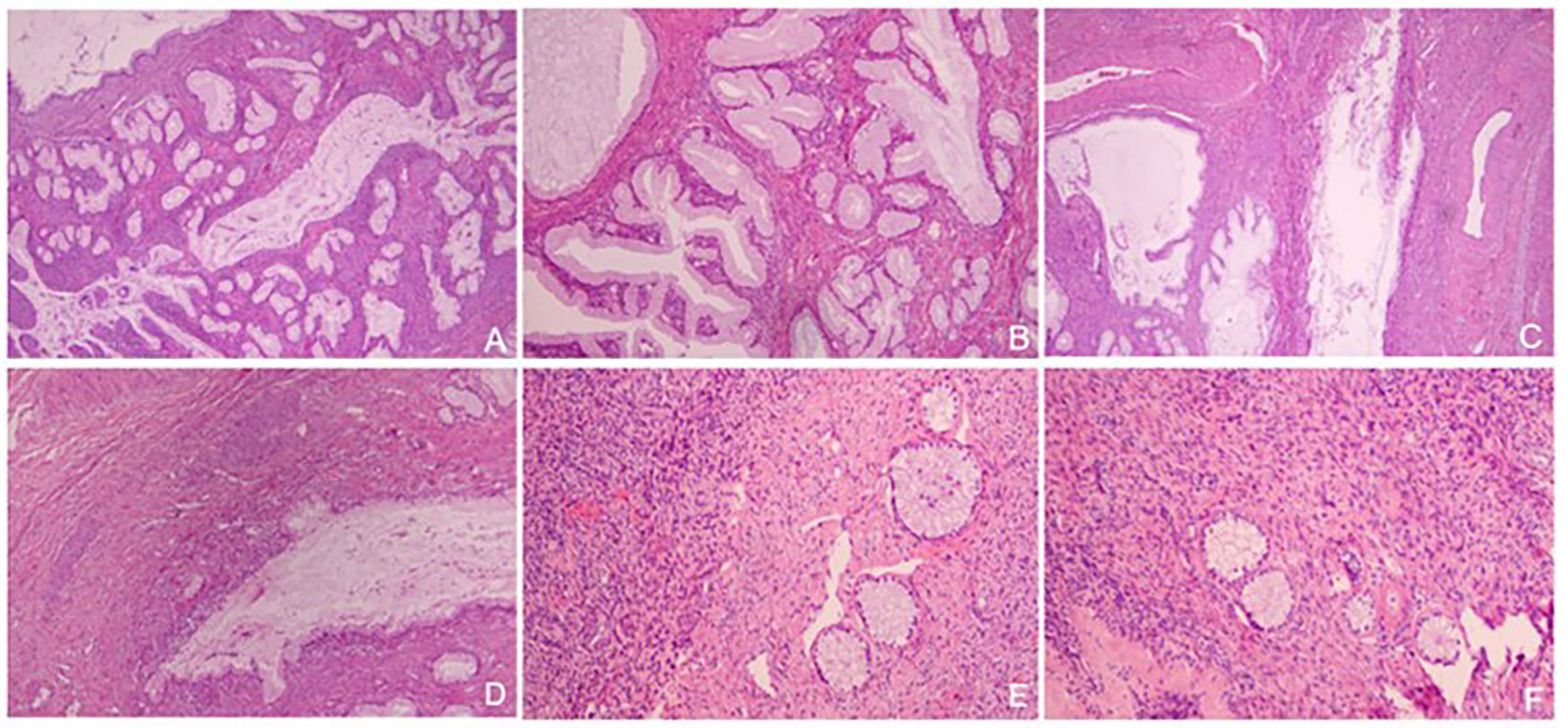
**(A,B)** Gastric-type endocervical adenocarcinoma (G-EAC) in cervix (H&E, × 40, × 100); **(C,D)** Deep stroma infiltrated with G-EAC (H&E, × 40, × 100); **(E,F)** The tumor cells of annular tubular cord tumors of the ovary are arranged into annular tubules, and the nuclei are polar overturned (H&E, × 100).

Overall, the patient’s cervix was infiltrated with the duct wall to > 2/3 of the deep myometrium and endometrium was unremarkable. Both ovaries were cystic and multilocular (approximately 4 × 3 × 1.5 cm; Figure 2D). Microscopic findings of the cervix were compatible with gastric-type endocervical adenocarcinoma. A variety of morphological glands were in the cervical interstitium (Figures 3A,B). Some of the glands were regular in morphology, some were like “chicken feet.” The cellular atypia was not obvious. The deep stroma was infiltrated with G-EAC (Figures 3C,D). As for SCTAT, the sex cord cells were arranged in a circular tubule. Besides, the nucleus was polarly reversed, close to the luminal surface with hyaline bodies around (Figures 3E,F).

**Figure 4:**
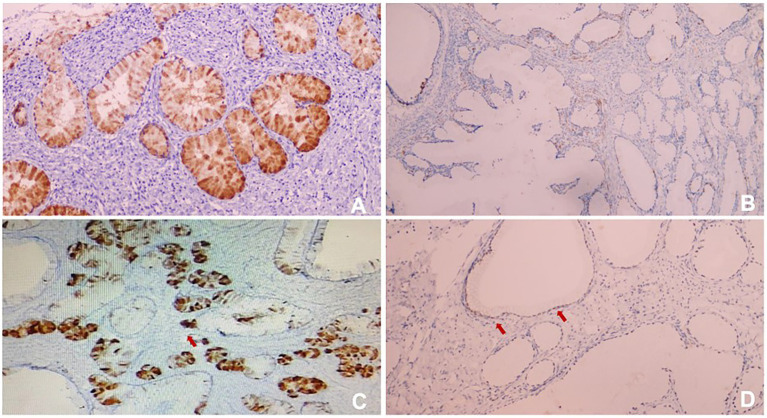
**(A)** HIK1083 (Positive); **(B)** P16 (negative); **(C)** Ki67 (60%positive); **(D)** p53 (wild type). All immunohistological (IHC) were × 100.

**Figure 5:**
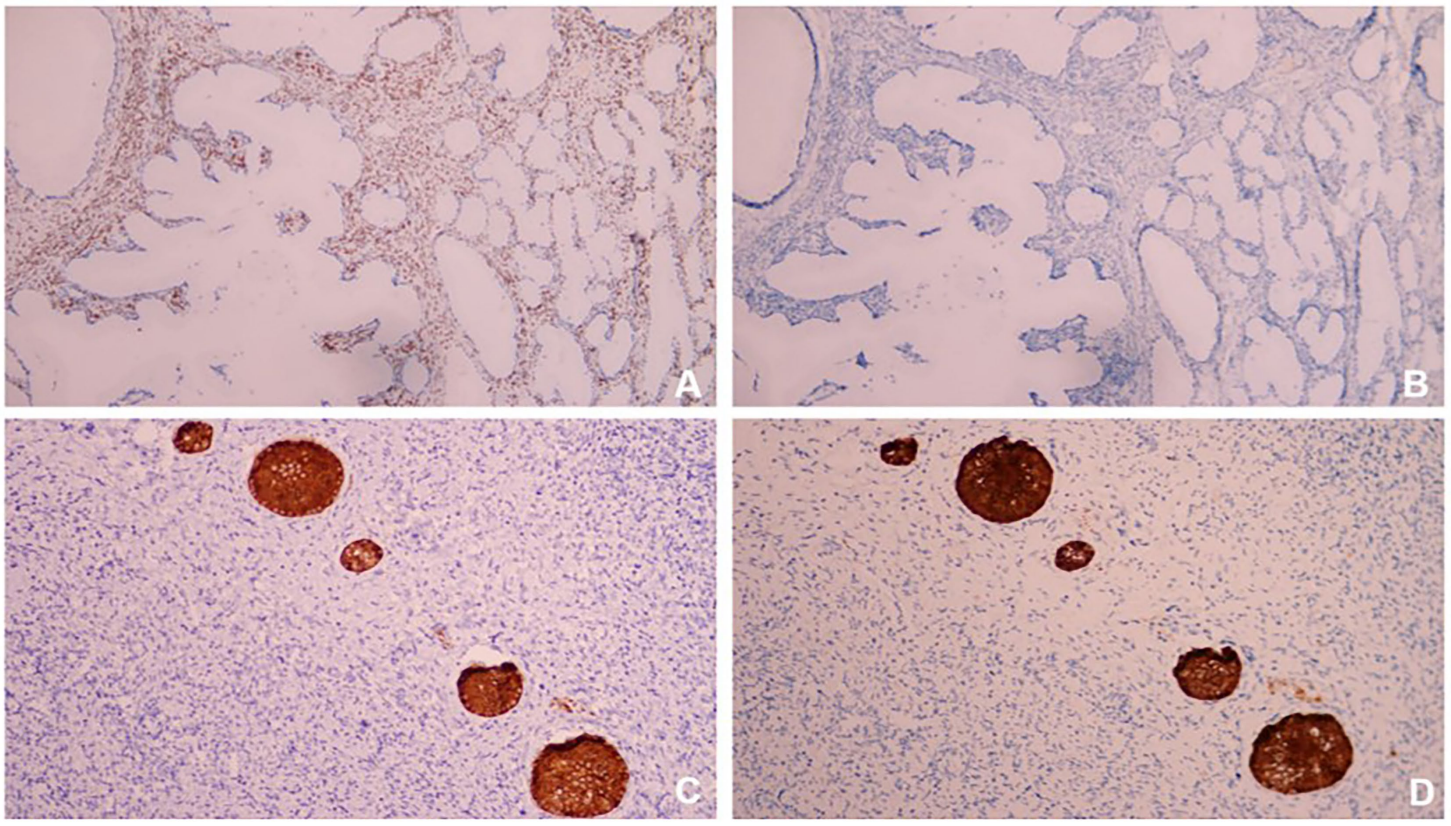
**(A)** ER (positive); **(B)** PR (negative); **(C)** Inhibin (positive); **(D)** Calretinin (positive). All IHC were × 100.

In order to confirm diagnosis, immunohistological (IHC) staining were conducted. The results showed (Figures 4, 5A,B): HIK1083 (Positive), P16(negative), Ki-67 (60% positive), p53 (wild type), ER (positive), PR (negative). Histologically, both cystically-dilated ovarian masses were confirmed to be SCTATs. IHC (Figures 5C,D): Inhibin (positive), Calretinin (positive). The pelvic lymph nodes did not show metastasis.

The patient received combined chemoradiation therapy with four cycles of Paclitaxel and Oxaliplatin. During the following 5 years of follow-up, the patient showed no evidence of tumor recurrence. However, the patient refused the STK11/LKB1 mutation test.

## 3. Discussion

Patients with PJS syndrome have a higher risk of developing gastrointestinal, breast, pancreatic, cervical, and ovarian cancers. For female patients, the risk of developing gynecological cancers is approximately 15 times higher than that of normal women (9). Recently, germline mutations in STK11/LKB1 were identified in more than 30% of patients with PJS (10). Therefore, once diagnosed, comprehensive gastrointestinal endoscopy, detailed family genetic counseling, and close lifelong follow-up are crucial. This article reviews the literature on the clinical features, diagnosis, treatment, and follow-up of PJS-related gynecological tumors. Taking this case as an example, we aimed to help the majority of clinical hospitals improve their understanding of the disease and avoid missing diagnoses and misdiagnoses.

### 3.1. Clinical features

The main features of PJS are multiple gastrointestinal hamartoma polyps, typical features of skin and mucous membrane pigmentation, and nonspecific clinical manifestations such as bleeding, intestinal obstruction, repeated intussusception, and intestinal obstruction caused by gastrointestinal polyps. Hamartoma polyps are most commonly found in the small intestine, followed by the jejunum, duodenum, and the colon. For patients with recurrent intestinal polyps, it is necessary to be aware of the risk of polyp carcinogenesis, which can gradually progress through the steps of hamartoma polyp-adenoma-adenocarcinoma (11). In addition, hypopigmentation of the skin and mucous membranes, which is widely seen on the lips, cheeks, and extremities, may first attract the attention of clinicians, and its dark or dark-brown spots are approximately 2–5 mm in size. Reviewing the medical history in this article, the patient had a history of partial resection of the small intestine due to intussusception. In addition, there were multiple pigmentation spots on the skin and mucous membranes, and multiple polyps in the gastrointestinal tract were found again by gastrointestinal endoscopy in the hospital, which strongly suggested the possibility of PJS.

The characteristics of patients with G-EAC can be summarized as atypical or nonspecific symptoms. Most of these patients complained of vaginal mucus or watery fluid and pelvic and abdominal masses. Unlike the HPV-related cervix with contact bleeding as the first symptom, this type of cervix is mostly smooth but enlarged, and the lesions are mostly located in the middle and upper parts of the cervical canal, forming a so-called “barrel-shaped” cervix (12). Some patients have cystic ovarian tumors, which are easily misdiagnosed as ovarian cancer.

### 3.2. Diagnosis

According to the WHO diagnostic criteria (13), the diagnosis can be made if any one of the following is satisfied: (1) three or more gastrointestinal polyps, and the smooth muscle originates from the muscular mucosa and extends to the submucosa; (2) positive family history; (3) pigmentation of skin and mucous membranes. The diagnosis of G-EAC mainly relies on pathological diagnosis combined with immunohistochemical staining if necessary. For the diagnosis of SCTAT, an ovarian tumor was found during the operation and the diagnosis was confirmed by postoperative pathology. In a case study of 74 patients with SCTAT, one-third had PJS (14). Therefore, for patients with clinically detected skin and mucous membrane pigmentation and symptoms such as irregular vaginal discharge, pelvic MRI should be completed in time, and if necessary, ultrasound-guided deep cervical biopsy, segmented curettage, and even cervical conization should be performed. The diagnoses of PJS and G-EAC complement one another. When a suspicious case is found clinically, one should be alert to the possibility of the other disease. Due to pigmentation of the skin and mucous membranes, this patient underwent partial bowel resection for intussusception when she was a child. The above two clinical features suggest that PJS is more likely to occur. In addition, pathology after cervical conization suggested a slightly deviated cervical adenocarcinoma. The patient was diagnosed with PJS with G-EAC.

### 3.3. Treatment and follow-up

At present, there is no guideline recommending a standard treatment regimen for G-EAC, so individualized treatment should be carried out based on the following cervical treatment norms. Surgery should be the primary treatment for patients with early stage G-EAC, and postoperative radiotherapy and chemotherapy ± targeted therapy should be used. Resection: attempt to remove all visible metastases. Patients with advanced disease are mainly treated with concurrent chemoradiotherapy. Studies have demonstrated that the expression of human epidermal growth factor receptor-2 (HER-2) is increased in G-EAC and is more common in patients with ovarian metastasis and advanced stage cancer (15). Thus, HER-2 monoclonal antibodies such as trastuzumab may become one of the targeted drugs for G-EAC. Currently, there is no radical cure for the treatment of PJS, mainly symptomatic and supportive treatment, and gastrointestinal endoscopy resection of multiple polyps is the first choice. There are few reports on SCTAT, and there is a lack of clear treatment guidelines; therefore, it is managed according to the treatment plan for sex cord-stromal tumors. Surgery, including total hysterectomy, bilateral adnexectomy, and omentectomy, is the mainstay for early stage patients. Since lymph node metastasis is the main route of SCTAT, pelvic and para-aortic lymph nodes should be removed simultaneously (16).

## 4. Summary

Gastric-type endocervical adenocarcinoma is a type of cervical mucinous adenocarcinoma, and its main characteristics can be summarized as follows: not related to HPV infection, low positive rate of cervical exfoliation cytology, typical symptoms and signs, and vaginal discharge. Its biological behavior is highly malignant, invasive, easy to transfer, easily drug-resistant, and has a poor prognosis. In addition to the characteristics of G-EAC, patients with PJS and G-EAC mainly exhibit skin pigmentation and multiple gastrointestinal polyps. Therefore, based on the clinical characteristics of these two diseases, clinicians and pathologists should increase their awareness and vigilance regarding the disease. Adequate physical examination, preoperative evaluation, and intraoperative exploration are important for the treatment and prognosis of patients. The sharing of this case is also aimed at enhancing obstetrics and gynecologists’ understanding of PJS and achieving early clinical detection and diagnosis, which is of great significance to patients and their families.

## Data availability statement

The raw data supporting the conclusions of this article will be made available by the authors, without undue reservation.

## Ethics statement

The study involving human participant were reviewed and approved by Medical Ethics Committee of Tianjin Central Obstetrics and Gynecology Hospital. The patient has provided her written informed consent to participate in this study. Written informed consent was obtained from the participant/patient(s) for the publication of this case report.

## Author contributions

XL: original draft writing. YQ: draft review and editing. WZ: picture presentation. YR: conceptualization. NZ: data curation. PQ: supervision. All authors contributed to the article and approved the submitted version.

## Funding

The study was supported by the Tianjin Science and Technology Plan Project (21JCQNJC00230).

## Conflict of interest

The authors declare that the research was conducted in the absence of any commercial or financial relationships that could be construed as a potential conflict of interest.

## Publisher’s note

All claims expressed in this article are solely those of the authors and do not necessarily represent those of their affiliated organizations, or those of the publisher, the editors and the reviewers. Any product that may be evaluated in this article, or claim that may be made by its manufacturer, is not guaranteed or endorsed by the publisher.
